# Addiction and Moralization: the Role of the Underlying Model of Addiction

**DOI:** 10.1007/s12152-017-9307-x

**Published:** 2017-02-19

**Authors:** Lily E. Frank, Saskia K. Nagel

**Affiliations:** 1 Department of Philosophy and Ethics, Technische Universiteit Eindhoven, P.O. Box 513, 5600 MB, Eindhoven, The Netherlands; 2 Department of Philosophy, University of Twente, P.O. Box 217, 7500 AE, Enschede, The Netherlands

**Keywords:** Addiction, Moralization, Moral responsibility, Stigma, Disease model

## Abstract

Addiction appears to be a deeply moralized concept. To understand the entwinement of addiction and morality, we briefly discuss the disease model and its alternatives in order to address the following questions: Is the disease model the only path towards a ‘de-moralized’ discourse of addiction? While it is tempting to think that medical language surrounding addiction provides liberation from the moralized language, evidence suggests that this is not necessarily the case. On the other hand non-disease models of addiction may seem to resuscitate problematic forms of the moralization of addiction, including, invoking blame, shame, and the wholesale rejection of addicts as people who have deep character flaws, while ignoring the complex biological and social context of addiction. This is also not necessarily the case. We argue that a deficit in reasons responsiveness as basis for attribution of moral responsibility can be realized by multiple different causes, disease being one, but it also seems likely that alternative accounts of addiction as developed by Flanagan, Lewis, and Levy, may also involve mechanisms, psychological, social, and neurobiological that can diminish reasons responsiveness. It thus seems to us that nondisease models of addiction do not necessarily involve moralization. Hence, a non-stigmatizing approach to recovery can be realized in ways that are consistent with both the disease model and alternative models of addiction.

## Introduction: Changing Models of Addiction

Despite the rich debate on a proper understanding of addiction, much of the oftentimes heated scholarly debate in numerous fields (medicine, psychology, philosophy) centers on whether addiction is a disease, i.e. a pathological compulsion the concerned cannot resist, or whether it is a matter of choice, i.e. a matter of willpower and self-control. The disease model of addiction taken broadly characterizes addiction as “severe, chronic stage of substance-use disorder, in which there is a substantial loss of self-control, as indicated by compulsive drug taking despite the desire to stop taking the drug“ [1; p. 364]. With a further focus on addiction as a brain disease, the National Institute on Drug Abuse (NIDA), characterizes addiction as

… a chronic, relapsing brain disease that is characterized by compulsive drug seeking and use, despite harmful consequences. It is considered a brain disease because drugs change the brain – they change its structure and how it works. These brain changes can be long lasting, and can lead to the harmful behaviors seen in people who abuse drugs [2 : p. 5]

Widely endorsed perspectives on addiction that follow the disease-model have held that addictive behavior is a compulsion – beyond one’s conscious control and without regard for one’s rational judgment – to indulge in particular behaviors or in the consumption of certain drugs [3, 4]. As even Lewis with a strong opposing perspective on the disease model recognizes [5#], “because addiction compromises our physical and mental health, and because it cannot be easily controlled, it seems like a disease” [p.##]. Those in support of addiction as a compulsion or chronic disease often point to the demonstrated role of genetic or neurophysiological factors in addiction, popularizing the notion of addiction as “hijacking the brain”.

Neuroscience and animal models of addiction have been particularly influential in this characterization. Identification of the neural pathways and circuits involved in addiction, particularly the mesolimbic reward system, and the ways in which the brains of addicted persons are similar and change in predictable ways is taken as significant evidence in favor of the disease model [4]. More evidence comes from the possibility to treat addiction through the use of pharmaceuticals to ease withdrawal and prevent relapses in drug and alcohol addiction [6]. Evidence also comes from animal research that shows rats and mice can become addicted to a variety of substances through repeated use and will engage in self destructive behaviors to access the substance [7, 8].

However, there are several challenges to the disease model of addiction and the subsequent compulsion thesis that follows from it. People who suffer from an addiction often refrain from engaging in addictive behavior for periods; and further, their addiction often requires an elaborate series of actions, which cannot all be compelled [9]. Some therefore argue that addiction is voluntary, a “disorder of choice“ [e.g. 10]. Addiction, like many basic choices that people make, is influenced by preferences and goals. The relief, if not pleasure, that is derived from satisfying one’s addiction could be understood as a rational choice. Moreover, Heyman [10] points out that available survey data indicate that most addicted persons eventually quit their addiction - data that is inconsistent with the chronic disease model [11]. Large-scale epidemiological studies show high percentages of spontaneous recovery, even without specific treatment [12; p. 169; 13, 14]. Stephen Morse illustrates this nicely:

The ability of many addicts to decide to quit and to be responsive to contingencies generally is an inconvenient fact for those who wish to conceptualize addiction as purely a brain disease. People do not stop being diabetics, for example, simply by deciding that their pancreases should produce more natural insulin nor does cancer abate because people have good reason to be free of this terrible disease [12; p. 169].

Finally, and perhaps most influentially, is a critique taken up by Marc Lewis [5]. While advocates of the disease model point to substantial changes in the brain that come with addiction, critics point out that the brain’s very nature is to change rather than remaining static. Changes to the brain do not necessarily indicate a disease process. Nor does the disease model alone seem to be an accurate depiction of the phenomenology/1st person accounts of addiction. Instead, there is evidence for a shifting role of pleasure in different stages of addiction, which at least suggests that someone suffering from addiction can be responsive to a variety of contingencies [15]. Notably, the models and the dominant discourses influence addicted persons’ reflections on the addict”s interior experience [16, 17]. Critics charge that a consequence of the disease model is that calling it a disease stigmatizes addicted persons, prevents them from developing self-control, and damages self-esteem.

The choice model, however, in turn does not remain without criticism, since there is evidence from cognitive neuroscience that the mechanisms involved in addiction are different from those engaged in ordinary choice. And choice does not adequately explain why addiction is a “chronic relapsing condition”, as it is often dubbed by health authorities [9]. Marc Lewis, in his *The Biology of Desire* [8] as well as in this volume [5] demonstrates how one can conceive of addiction as related to significant brain changes and *at the same time* understand how addiction is “motivated repetition that gives rise to deep learning” [5]. This ultimately allows for attributing self-control and the capacity to learn otherwise to those who previously learned addictive habits. Lewis and other critics of the disease model address worries that alternative models will blame addicts, thrust unwarranted moral responsibility on them, and remoralize addiction.

## Does the Rejection of the Disease Model Risk the (Re-) Moralization of Addiction?

Instead of arguing whether addicted persons can make volitional choices or are out of control, and whether they are to be held responsible for their addiction and/or for their actions, in this article, we would like to focus on a different set of questions: We would like to shed light on the relation between addiction and moralization. Morality and addiction are often entwined. We investigate which factors of the different models foster or hinder moralization. What role does our understanding of moral responsibility play for moralization of addiction? What is the reason that addictions come to be treated as matters of morality, subject to moral evaluation, responsibility, and blame? What are the consequences of the moralization of addiction?

### Moralization – What Do We Mean by It and Why Does It Matter?

The past two centuries of the history of Western developed countries public health efforts are rife with examples of the process of moralization (and de-moralization). Cigarette smoking, the consumption of high fat foods, becoming obese, entering environments rich with infectious microbes (as in pregnant woman travelling to a Zika infected country or an elder care provider failing to get a flu shot and going into a school during flu season) have all at one point in recent history shifted from being merely individual preferences to taking on not only a negative valence when it comes to health, but a further negative moral connotation as well [19–23].

Moralization refers to conversion of a preference into a value, within a culture and in individual lives. It is hypothesized that values, because of associated moral meanings are more likely to produce internalization than instrumental concerns such as health risks. Specifically, it is predicted that liking for and disgust towards a substance or activity will be more extreme if the substance or activity is treated as a value (is moralized) [24; p. 321].

We would like to specify this definition by focusing on *moral* values, as opposed to economic or aesthetic values (although they are often related). In principle, moralization need not always associate behaviors or preferences with *negative* moral meanings. Something like exercising regularly to maintain cardiovascular health can be moralized in the sense that a person who does this can be understood as engaging in more than a health promoting behavior, but also a virtuous or dutiful behavior or lifestyle as well. In the majority of cases discussed however, when something becomes moralized, it takes on a negative moral meaning. Most often, moralization also involves a shift in focus to the individual as the problem, not the phenomenon itself, nor the social context.

It is necessary here to distinguish moralization in the sense we are using it, which involves attributing moral weight or significance to some act or practice, from a related but distinct use of moralization or moralize associated with self-righteous judgmentalism.^1^ People who constantly and wrongly attempt to turn trivial matters into ones apparently involving morally significant features are engaged in this kind of moralism.

We would like to stress another important distinction: Whether or not a practice becomes moralized is an empirical, psychological, anthropological, and sociological issue. We are interested in the normative question; that is, whether or not something *should* be moralized, which matters for several reasons

First, it may cause a shift in focus from social and economic causes of disease or other kinds of problems to a focus on individual responsibility exclusively.^2^ Deborah Lupton has pointed out the way in which self-tracking technologies encourage this perspective, encouraging certain health problems to “become represented primarily as failures of individual self-control or efficiency, and therefore as requiring greater or more effective efforts, including perhaps increased intensity of self-tracking regimens, to produce a ‘better self.’”[26; p. 7]. The upshot of this can mean different kinds of policies, levels of social tolerance, and distribution of resources in ways, which may be unjust.

Secondly, moralization is a significant phenomenon from the perspective of normative ethics – not only from sociology, public health, etc. In fact it is at the heart of one of the most fascinating parts of human moral and ethical practice. We have an ability to pick out a certain phenomenon, behavior, or traits as morally relevant and others as not so. Without a very long and complicated story: the color of socks that one chooses to wear in the morning is taken to be a morally neutral decision. But a decision about whether or not to give one’s child breakfast or let it leave the house hungry is usually taken to be a morally fraught decision, whatever the mitigating circumstances might be. In this case, moralization of an activity is closely related to the extent to which a person should be held morally responsible for their condition. So it matters to the extent that we want to make attributions of moral responsibility only when they are justified. Moralization matters if we care about consequences of attributing moral responsibility regardless of whether the attribution is justified or not.

Consider the recent modern history of western societal views on homosexuality: once considered immoral, then as a disease, the moral implications shifted. Notably, in this case, the negative moral patina remained (at least for some) despite the medicalization. With the process of de-medicalization, moral attitudes might be influenced as well. Examples of other cases in which new scientific insights on the health consequences of a behavior lead to moralization include cigarette smoking, and obesity and healthy eating [23, 27, 28,]. Rozin [29] describes the history of societal attitudes toward cigarette smoking as the “quintessential example” of moralization. In many Western countries, cigarette smoking has changed over the past 50 years from a preference to a moral violation, as its health effects became better known. Cigarette smoking has now become moralized to such an extent that disgust reactions towards smoking correlate more highly with negative moral judgments of smoking than do the negative perceptions regarding health risks [24].

### Does the Disease Model Reduce the Entanglement of Moralization and Addiction?

Addiction appears to be a deeply moralized concept. Addiction is as much understood to be about physiological and psychological processes, just as it invokes moral discourse. The position towards it can involve condemnation of supposed excess, in the USA in the 1970s it lead to the disputed “war on drugs“.

We are interested in examining the ways in which addiction (specifically substance abuse addictions) may become increasingly moralized (again) as a result of challenges to the brain disease model of addiction. Importantly, we ask whether or not moralization is an inevitable with the rejection and replacement of the brain disease model and whether or not this is a morally desirable consequence. It seems that once a condition is conceived of as a medical condition, its part in the moral discourse lessens or at least changes significantly. Once addiction is understood as a brain disease, questions about right- or wrong-doing seem to be ill-posed. As soon as something is pathological, the main concern is about how to cure it. The moral questions that do remain regard the extent to which we should invest scarce medical resources in its treatment and prevention, for example. Moral issues arise with many illnesses. Consequently, one needs to ask if one criticizes the disease model, and proposes a model where either choice or learning play a central role, does that automatically lead to moralization of addiction?

What are the advantages and disadvantages of moralization of addiction? What effect is this likely to have on the way we conceive of individual and social responsibility, blame, autonomy, justice, and on views of the good life in both the public and the private sphere?

Is the disease model the only path towards a ‘de-moralized’ discourse of addiction? It is tempting to think that medical language surrounding addiction provides liberation from the moralized language. But this is not necessarily the case. The disease model of addiction has not silenced the moralized discourse. At least it seems to be clear that the story is not that simple: Moralization of addiction did not vanish after addiction became widely discussed in terms of disease. This is evident in the many biases and negative social and health-related consequences that addicted persons continue to face. These are harms that result, not directly as a result of their behavior or substance consumption, but as a byproduct of how other people marginalize them or treat them differently. Moreover, it might be that the disease model invites its own forms of moralization: The conception of addiction as a disease, suggesting that people cannot take control of it, might invite morally laden perspectives and possibly stigmatization on it, exactly because it is understood as a disease [30].

### The Dangers of Moralization

What can explain the fact that despite the official dominance of the disease model of addiction, addiction remains moralized in many ways? One possibility is that we can simultaneously hold two contradictory attitudes about addiction at the same time or may hold that addiction is a combination of a disease and a moral failing, or perhaps even a disease caused by a moral failing. But there are other explanations as well. Other factors, such as social marginalization of certain groups of addicts (especially users of hard drugs) [31–33] and the mere fact that addictions have harmful effects (regardless of whether there is volitional choice involved) are likely having a role as well. In some contexts the extent to which certain types of addiction are moralized and stigmatized is connected to race. Reeves and Campbell [34] discuss this phenomenon in their work on media depictions in the United States of users of smoked cocaine, so-called crack, associated with African American users and powdered cocaine, associated with white users.

Empirical studies of the social perception of addictive disease are rare. Shaffer [35] found a distinction between the perception of biological (amongst others described as tuberculosis, coronary thrombosis, asthma) and behavioral disorders (amongst others described as cocaine abuse, heroine dependence, alcohol abuse, addiction). A recent review on stigma and marginalization related to psychoactive substance use [36] found ongoing social disapproval, and there is evidence for a stronger desire for social distance towards alcohol dependent persons than towards those with psychiatric diagnoses as depression or schizophrenia [37].

It is not only the general public that has negative moral feelings towards addicted persons. There is widespread evidence that health care providers share these biases - resulting in care that is either deficient, lacking in caring attitude, or involves extensive judgment. The phenomena of physician biases, distancing, and reluctance to treat, certain groups of people has been studied extensively, including people who are obese, have mental health disorders, substance abuse disorders, and eating disorders [38]. Research comparing the attitudes of the general public and physicians to alcohol addiction found that these two groups did not display distinct stigmatizing attitudes on alcohol addiction, in fact; “2 out of 3 people in both groups have negative attitudes towards alcohol addicts and medical education did not change these attitudes” [39] Medical students, for example, display clear preferences for particular categories of patients, and were less sympathetic toward those whom they believed to be undeserving of treatment because they were responsible for their condition, for example people with eating disorders [40] and the obese [41, 42].

Recent evidence suggests that the way we talk about addiction matters. Language that treats it as a disease or a moral failing has a significant impact on health care providers’ attitudes towards those patients [43] Physicians were given vignettes about a patient. There were two versions of the vignette, one in which the patient is described as a “substance abuser” and in the other as “someone with a ‘substance use disorder’.” When participants received the vignette describing the patient as a substance abuser their moral evaluations of the patient changed- they “were more likely to agree with statements that he was to blame for his problem and should be punished for not adhering to the court-ordered treatment program, when compared with respondents whose surveys described him as someone with a substance abuse disorder” [44].

There is extensive evidence that shows that when physicians hold beliefs about addiction that moralize the condition or stigmatize the addict, this seems to create several barriers to getting sufficient care from health care providers [45–50]. When patients perceive this attitude they are less likely to be open and honest about their habits and perhaps will avoid seeking care altogether. Physicians with these kinds of negative attitudes may also fail to address substance abuse issues with their patients altogether because of personal discomfort. The data supports the idea that public and professional perceptions maintain the view that addiction results from personal choice and immoral values, conceiving of addiction as different from biologically based diseases, despite their perception of it as a disease.

## Alternatives to the Disease Model and their Treatment of Moralization and Moral Responsibility: Owen Flanagan, Marc Lewis, and Neil Levy

The above findings can in part support the normative claim that further moralization of addiction is undesirable when aiming to provide help to people who are suffering from it. We now turn to the question of whether or not alternatives to the disease model of addiction necessarily involve further moralization of addiction. The philosophical literature on moralization as such, and specifically on moralization of addiction related to alternatives to the disease model is limited. Therefore, we discuss the thoughts of three critics of the disease model, who propose alternative views, and explicitly address this issue. The following is not meant to provide a complete discussion of addiction and moral responsibility, which would be far beyond the scope of this paper [See 59]. Rather, we seek to illustrate the ambiguity in whether or not alternative models of addiction involve re-moralization.

### Owen Flanagan’s Twin Normative Failure Model, Marc Lewis’s Habit and Deep Learning Model, and Neil Levy’s Defect of Agency Model

Owen Flanagan theorizes addiction as a “twin normative failure.“ By this he means that:

A failure of normal rational effective agency or self-control with respect to the substance; and shame at both this failure, and the failure to live up to the standards for a good life that the addict himself acknowledges and aspires to [51; p. 1].

Flanagan’s brief dismissal of the re-moralization objection cannot be viewed in isolation from his views on addiction and moral responsibility. In an elaboration entitled “Responsibility without the sting”, he relies on Strawson’s account of reactive attitudes and objective attitudes, where reactive attitudes are reactions based upon an interpretation of conduct, manifested in actions and attitudes, and objective attitudes are those seeing others as objects of a handling, as subjects for treatment, not as freely acting moral agents. Flanagan tentatively claims we should suspend reactive attitudes toward addicted persons, and addicted persons should suspend them when they reflect on themselves [51; p. 7]. He argues we are justified in reacting to them in a way similar to the way we react to people “who have no rational control over their actions” - children or the “insane” as he puts it. But, crucially, he is not arguing that addicted persons have no rational control over their behavior. Although this suspension of the reactive attitudes is difficult to assume with regard to addicted persons, he is optimistic that:

The more we learn about the complex socio-psycho-biological nature of addiction, about the ways various cultures encourage heavy drinking, about the effects of SES and drug availability about genetic propensities, about the effects of weird reinforcement regimens, and of brain glitches, we have reason to adjust full normal subjective engagement to the addict [ibid; p. 7].

Flanagan also directly responds to the criticism that his account of addiction is a way of “re-moralizing.” It is appropriate that the addict feels shame, he argues, because they are reflecting on their inability to act as “an effective agent in relation to the Substance” [ibid; 10]. He concludes that, we should “accept that addiction just is a normative disorder, while at the same time not moralizing it” [ibid].

These are, however, very limited responses to the worry about re-moralization. If, as he recommends, we should take the objective attitude towards addicted persons and suspend the reactive attitudes it is important to be very clear about why we should do so. In the cases of the insane and children we do this because, as he says, we believe they are not in control of their actions. But this is precisely one of the elements of the disease model of addiction that Flanagan wishes to challenge with his normative failing model that characterizes addiction as a choice. Hence, this cannot be the reason. We are left with the possibility that addiction, although a choice, is a condition where there is mitigated responsibility to some extent.

Alternatively, it could be that in cases of addiction there are other mitigating reasons that make addiction excusable. That is, even though the addicted persons are responsible, and feelings of shame are warranted, they are not truly blameworthy. This seems to be what Flanagan is suggesting when he says that as we learn more about the nature of addiction and its complex causal trajectory, we may mitigate the extent to which we morally engage with the addict in the same way we do with non-addicted persons. In order to flesh this out, Flanagan (or someone else) needs to give an account of what the excusing reasons are in the case of addiction-reasons that are consistent with his model.

In another challenge to the disease model, Neil Levy argues that instead of a brain disease, addiction is better understood as a “disorder of belief” [52]. Levy is explicitly interested in issues of moral responsibility and by association the moralization of addiction [58]. He offers a complex picture of the causes of addiction and its maintenance putting strong emphasis on the “social conditions” outside of the individual addicted person’s control that contribute to their state.

Addiction is not a brain disease, but there is a good case for saying that it is, nevertheless, a disorder which may require treatment (which may be medical or psychiatric, though other kinds of treatment may be appropriate in addition or instead), for which the sufferer is not to blame and the sufferer from which is an appropriate recipient of compassion [53; p.6]

Levy also rejects the “crass moralism” of the pre-disease model days in addiction science, where the addict was blamed, ostracized, and isolated. He argues that a rejection of the brain disease model does not mean we return to this. Instead, a view of addiction that takes social circumstances into much greater account may have the opposite effect: society, professionals and organizations who think about addiction will have to take into account a much wider range of factors than simply brain dysfunction when considering the causes, preventives, and solutions for addiction.

For Marc Lewis, addiction is neither a disease nor a voluntary choice. Instead he characterizes it as

…a habit that grows and self-perpetuates relatively quickly, when we repeatedly pursue the same highly attractive goal. Or, in a phrase, motivated repetition that gives rise to deep learning [18; p. 174].

Lewis pays attention to the neuroscientific evidence regarding brain changes that come with addiction and argues that if we take changes to “the wiring of the striatum (and related regions)” and adjustments in “the flow and uptake of dopamine” to be evidence of a disease in the case of addiction, then we also should count the habits that lead us to repeatedly seek rewarding experiences, like meeting a lover, as disease processes because they involve the same structural and functional changes [18]. The process of becoming addicted can be described as a process of deep learning that is fuelled by desire and becomes a habit [18].

Like Flanagan and Levy, Lewis is aware of the worry that rejection of the disease model of addiction might be an expression of or will result in further stigmatization of addicted persons and an attitude that we as a society should leave them to their own devices, because it is not as if they have a “real disease” like cancer or diabetes. Importantly, he states that “I’m not arguing that addictive behaviors are fuelled by *voluntary* choice” [5]. The key here is the notion of voluntary. Lewis argues that it is perfectly consistent to say that addiction is fueled by desire and choice, but that not all choices are created equal:

The leak in the logic is the assumption that choice is a deliberate, rational function we can apply at will. But choice is nearly always irrational—which is only to say that it is executed by the same brain that gives rise to hope, need, fear, and uncertainty, a brain that’s highly sensitive to learned associations and contextual cues, a brain that forges new connections based on the activation of existing connections and the strong emotions they render. [5]

When addressing one of the purported benefits of the disease model of addiction, i.e. that it frees us from “denigrating addicts for their lack of willpower and moral decrepitude” Lewis [18] argues that “[d]espite the despicable things addicts sometime do, intense shame and guilt are more likely to thwart recovery than facilitate it” [5]. So as a practical matter as well, blaming addicted persons is not a good way to enable their recovery [51].

## Moral Responsibility - a Key Issue for Moralization

The debate about addiction in terms of compulsion versus choice has an important impact on whether, or to what degree, we (should) hold addicted persons morally responsible for their addictions and for actions resulting from their addictions. The reasoning seems simple: For a person to be responsible for her actions, she has to be in control of them. Following the disease model, control over one’s addictive behavior is largely absent, and thus for the most part one cannot be held responsible for it. On the choice model, however, addicted persons use the same assessment and volition mechanisms as non-addicted persons, which would suggest they are largely in control of their actions, and therefore responsible for their addictive behavior. However, just as the complex addiction comes in degrees, so do the different facets related to it such as control and responsibility [54]. Neither the complex nor the facets are absolute, but they come on a spectrum. Moreover, whether one is held responsible for one’s addictive behavior often also depends on whether one has sought help and tried to quit.^3^

Those viewing addiction in terms of choice may have reasons to consider its negative effects as a matter of responsibility and blame, although it may only be part of the explanation for why addictive behavior is frequently the subject of moral indignation. Notably, the two camps sketched by now are usually seen as incompatible: If addiction is a disease, then there is no place for choice and self-control. If it is a matter of choice, then the concerned are to be held responsible for it – with all the social, legal, and health consequences there are.

A key concept related to moralization is moral responsibility, along with the accompanying emotions of guilt, shame, and blame. A widely shared criterion for moral responsibility is that the agent is not compelled or that he is free in the relevant sense.

As a behavior or state becomes moralized, we increasingly assume that the individual is morally responsible for finding themselves in that state or the consequences of their behavior or state. There is a vast debate on moral responsibility and addiction and we will not cover in any detail here [but see 56–60]. We also wish to further narrow our focus to the choice to continue to engage in the addictive behavior or consume the addictive substance as the act in question, rather than other morally problematic acts the addict may perform as a result of their addiction or the choice to begin using the substance in the first instance. In this section we highlight an example of the type of conditions taken to signal that someone is morally responsible. We add the example of the reasons responsiveness view to show that while the conditions for just this one aspect of moral responsibility are complex in the absence of the disease model, they are also complex and difficult to apply given the disease model.

Sinnott-Armstrong focuses on the diversity among addicted persons and then asks the question of whether or not there is a unifying characteristic among them. That would make us wonder whether or not they are responsible for their behavior and in what sense they may not be “in control” of their behavior in the way that non-addicted persons are [54; p.122]. He argues that a good way to understand control in this context is to study reasons responsiveness in line with the position of Fischer and Ravizza [61]. In other words, an agent has control when, in simplified terms, the agent usually is receptive to and acts based on the reason he or she has or the reason he or she believes she has [54; p.130; 62].

On this view different addicted persons are in control of their behavior to different extents in different circumstances, because they are capable of responding to reasons to differing degrees depending on the context, for example a cocaine addict may not be responsive to her reasons not to use the drug in social situations in which she feels uncomfortable [54; p. 135]. Similarly they can he held morally responsible to various degrees, depending upon other things, on the extent to which they were in control of their actions.

### Connecting Reasons Responsiveness to Moralization and Addiction

Lack of control in the form proposed by Sinnott-Armstrong and Fischer and Ravizza as a lack of reasons responsiveness cannot tell us definitively whether addicted persons as a class of persons are responsible for continuing to use their substance. What is relevant here is that a deficit in reasons responsiveness can be realized by multiple different causes, disease being one, but it also seems likely that the alternative accounts of addiction given by Flanagan, Lewis, and Levy, may also involve mechanisms, psychological, social, and neurobiological that can diminish reasons-responsiveness. It thus seems to us that non-disease models of addiction do not necessarily involve moralization. Notably, for some there is an explicit effort to avoid this as a consequence of their theory. While some who endorse alternative models of addiction also make claims about the moral responsibility of addicted persons, none of them give an all or nothing answer. Usually the answer is: to some extent and in a mitigated way.

## Outlook

On first assessment it might seem obvious that a rejection of the disease model of addiction necessitates a re-moralization of addiction – a return to blaming, shaming, and perhaps even punishing addicted persons for their substance seeking and consuming behavior. We aimed to dissect how different models of addiction foster or hinder moralization and argued that the diversity of forms of addiction does not allow a one-size-fits all answer.

Building on accounts of moral responsibility, we argued that a deficit in reasons responsiveness can be caused by a disease but also by diverse psychological, social, and neurobiological mechanisms. Notably, non-disease models of addiction do not necessarily involve moralization.

From a normative perspective the entwinement of addiction and moralization is worrisome for two central reasons. The first is that moralization and the accompanying stigma persists despite the prevalence of the disease model of addiction, causing harm to addicted persons and society at large. We predict with concerns that greater embrace of this stigmatization would lead to even greater harms and steps backward in the social practices, laws, and public health methods surrounding addiction. Secondly, moralization itself is an important phenomenon for normative ethics. In the case of addiction it is closely related to the concept of moral responsibility and may also be for other health conditions that run the risk of becoming moralized or have been moralized in the past - such as obesity or depression.

Future research on the relationship between models of addiction and moralization will be further complicated by the addition of the concept of medicalization. We wish to investigate the ways in which medicalization and moralization differ and overlap, both conceptually and in terms of their recent social trajectories. Relatedly, stigma is often part of negative moralization, but can also come along with medicalization. We suggest looking into the ways in which models of addiction may exacerbate or reduce stigmatization for certain groups of addicted persons. Further empirical and conceptual research is needed on the ways in which different models of addiction may impact addicted persons’ quality of life and quality of health care, including public health research. Finally, consideration should be given to the way the models potentially alter the self-understanding of addicted persons, relying on phenomenological accounts. Ideally, those efforts combined with raising awareness of the moralization of addiction ultimately can lead to an approach to recovery in a non-stigmatizing way.

## Footnotes

1 Thank you to our anonymous reviewer for suggesting we clarify this point.

2 Elsewhere, one of the authors has discussed some of the consequences of such a shift of focus on perceived responsibility [25].

3 For a closely related discussion see Hannah Pickard in [55].
